# A Rare Case of Appendiceal Goblet Cell Adenocarcinoma Complicated by Development of a Loculated Pleural Effusion

**DOI:** 10.7759/cureus.111922

**Published:** 2026-07-01

**Authors:** Elena Arcaroli, Adam Hurwitz, Timothy Johnson, Mitul Patel

**Affiliations:** 1 Family Medicine, Nova Southeastern University Dr. Kiran C. Patel College of Osteopathic Medicine, Tampa, USA; 2 Internal Medicine, HCA Florida Healthcare/USF Morsani College of Medicine GME, HCA Florida Largo Hospital, Largo, USA; 3 Internal Medicine, Lakeland Regional Health, Lakeland, USA

**Keywords:** appendiceal goblet cell adenocarcinoma, goblet cell adenocarcinoma, loculated pleural effusion, rare abdominal tumor, rare appendiceal pathology

## Abstract

Appendiceal goblet cell adenocarcinoma (GCA) is a rare malignancy that often presents as acute appendicitis. We present a 57-year-old woman with a history of breast cancer who presented with right lower quadrant pain, fever, nausea, and vomiting and underwent appendectomy for presumed uncomplicated appendicitis. She was discharged postoperatively, and final pathology subsequently revealed GCA of the appendix. Approximately one week later, she returned with dyspnea and was found to have a new, multiloculated, exudative pleural effusion. Pleural fluid cytology was negative for malignancy. She required chest tube placement with intrapleural fibrinolytics followed by surgical decortication for definitive management. Given recent surgeries and concern that early positron emission tomography/computed tomography (PET-CT) would be confounded by postoperative inflammation, a multidisciplinary decision was made to initiate systemic chemotherapy prior to right hemicolectomy. She later underwent hemicolectomy with a favorable outcome and no evidence of peritoneal metastasis. This case highlights an unusual extraperitoneal manifestation of appendiceal GCA and an individualized treatment sequence differing from standard management, underscoring the need for further study of optimal treatment sequencing and surveillance strategies.

## Introduction

Appendiceal goblet cell adenocarcinoma (GCA) is a rare neoplasm with features of both adenocarcinomas and neuroendocrine tumors (NETs), affecting approximately 0.01 to 0.05 per 100,000 people per year [1]. It has an average age of presentation of 52-58 years, with a predisposition for White populations and conflicting data regarding sex predilection [1,2]. Reported associations include ulcerative colitis, neurofibromatosis type 1, and ovarian mucinous cystadenocarcinoma [3]. Schistosomiasis has also been described as a potential risk factor [1]. 

GCA is predominantly comprised of goblet-like mucinous cells with some endocrine and Paneth-like cells, although its histogenesis and exact cell of origin remain incompletely understood [2]. The classification of this entity has evolved over time, and the World Health Organization (WHO) now designates it as appendiceal GCA, recognizing it as a distinct pathological entity separate from neuroendocrine tumors and conventional adenocarcinomas [4]. For consistency with the majority of existing clinical literature and prior publications, the term GCA will be used throughout this report.

Most cases (65-67%) of low-grade GCA present with signs and symptoms of acute appendicitis, including nausea, vomiting, leukocytosis, and right lower quadrant abdominal pain [2]. In these patients, GCA is often found incidentally upon postoperative microscopic examination of the appendix [2]. Other clinical presentations include abdominal pain (20%), abdominal distention (18%), and bowel obstruction (10%) [1]. These symptoms are frequently related to metastatic disease, most commonly involving the peritoneal cavity [2,5]. GCA characteristically demonstrates a propensity for transcoelomic spread, with peritoneal dissemination and ovarian involvement being the most common patterns of metastasis, while hematogenous and distant extraperitoneal spread are less frequently observed [2,5].

Large case series and systematic reviews describe the peritoneal cavity, ovaries, liver, and small bowel as the most common sites of metastasis, reflecting the tumor’s tendency for intraperitoneal dissemination [2,5,6]. In contrast, thoracic involvement is rarely reported. When present, it is typically associated with advanced metastatic disease rather than isolated inflammatory processes [6]. Notably, existing literature does not describe multiloculated inflammatory pleural effusions occurring in the absence of metastatic thoracic disease, highlighting an atypical extraperitoneal manifestation of GCA [6,7].

Right hemicolectomy is the recommended first-line treatment for GCA in most cases. One exception includes tumors <1 cm confined to the appendix without high-risk features, in which case appendectomy alone may be therapeutic [8]. Adjuvant chemotherapy is recommended in patients with stage III and IV disease, as well as in recurrent disease [8]. Although there are no randomized controlled trials for the choice of systemic chemotherapy, adjuvant chemotherapy is typically based on a fluorouracil (5-FU) regimen due to the resemblance of metastatic GCA to conventional colonic adenocarcinoma [8]. Additionally, retrospective studies suggest that cytoreductive surgery with hyperthermic intraperitoneal chemoperfusion (CRS/HIPEC) may improve median survival in select patients from 18 to 37 months, and 4-year survival rates up to 24% [5].

Prognosis is primarily based on stage at diagnosis and grade of tumor [9]. Early stage disease is often associated with favorable outcomes, while advanced stages carry a worse prognosis [10]. Histologic grade has also been shown to correlate with overall survival independent of stage, with the high-grade adenocarcinomatous component being a key determinant of prognosis [9,11].

To our knowledge, isolated extraperitoneal thoracic manifestations of GCA without evidence of metastatic disease are rarely described, and multiloculated inflammatory pleural effusions have not been previously reported in this context [6,7]. This case highlights an atypical clinical presentation as well as a deviation from the standard treatment sequencing, underscoring gaps in the current literature regarding thoracic complications and individualized management strategies in GCA.

## Case presentation

A 57-year-old female with a history of breast ductal carcinoma in situ (DCIS) status post bilateral mastectomy and tamoxifen therapy presented to the emergency department (ED) with sudden-onset, sharp lower abdominal pain associated with decreased appetite, fever (102.8°F), nausea, and vomiting that awoke her from sleep. 

Initial blood work was notable for a leukocytosis of 12.1 x 10^9^/L and a lactic acidosis of 2.2 mmol/L, which improved with intravenous fluid resuscitation. Physical examination revealed abdominal distention with right lower quadrant tenderness and rebound. Computed tomography (CT) of the abdomen and pelvis demonstrated acute appendicitis with concern for perforation, as well as a small right pleural effusion. A chest radiograph (CXR) obtained at that time did not demonstrate a pleural effusion or other acute cardiopulmonary process. The patient was started on intravenous piperacillin-tazobactam and underwent urgent laparoscopic appendectomy. Intraoperative findings were notable for a perforated appendix with localized inflammation. Her postoperative course was uncomplicated, and she was discharged the following day on a seven-day course of amoxicillin-clavulanic acid with general surgery follow-up.

Final pathology from the appendectomy, reported six days postoperatively, revealed GCA of the appendix (grade 2, moderately differentiated, pT3) with tumor invasion through the muscularis propria into the subserosa and negative margins. Lymphovascular and perineural invasion were not identified.

Approximately one week after discharge, the patient returned to the ED with progressively worsening fatigue, shortness of breath, and right-sided chest pain radiating to the back. Previous CT imaging had demonstrated a small right pleural effusion that was not appreciated on CXR, which had significantly progressed by the time of readmission. On examination, she was hemodynamically stable with decreased breath sounds in the right middle and lower lung fields. CT angiography of the chest demonstrated a large, multiloculated right pleural effusion with associated basilar consolidation and no evidence of pulmonary embolism.

**Figure 1:**
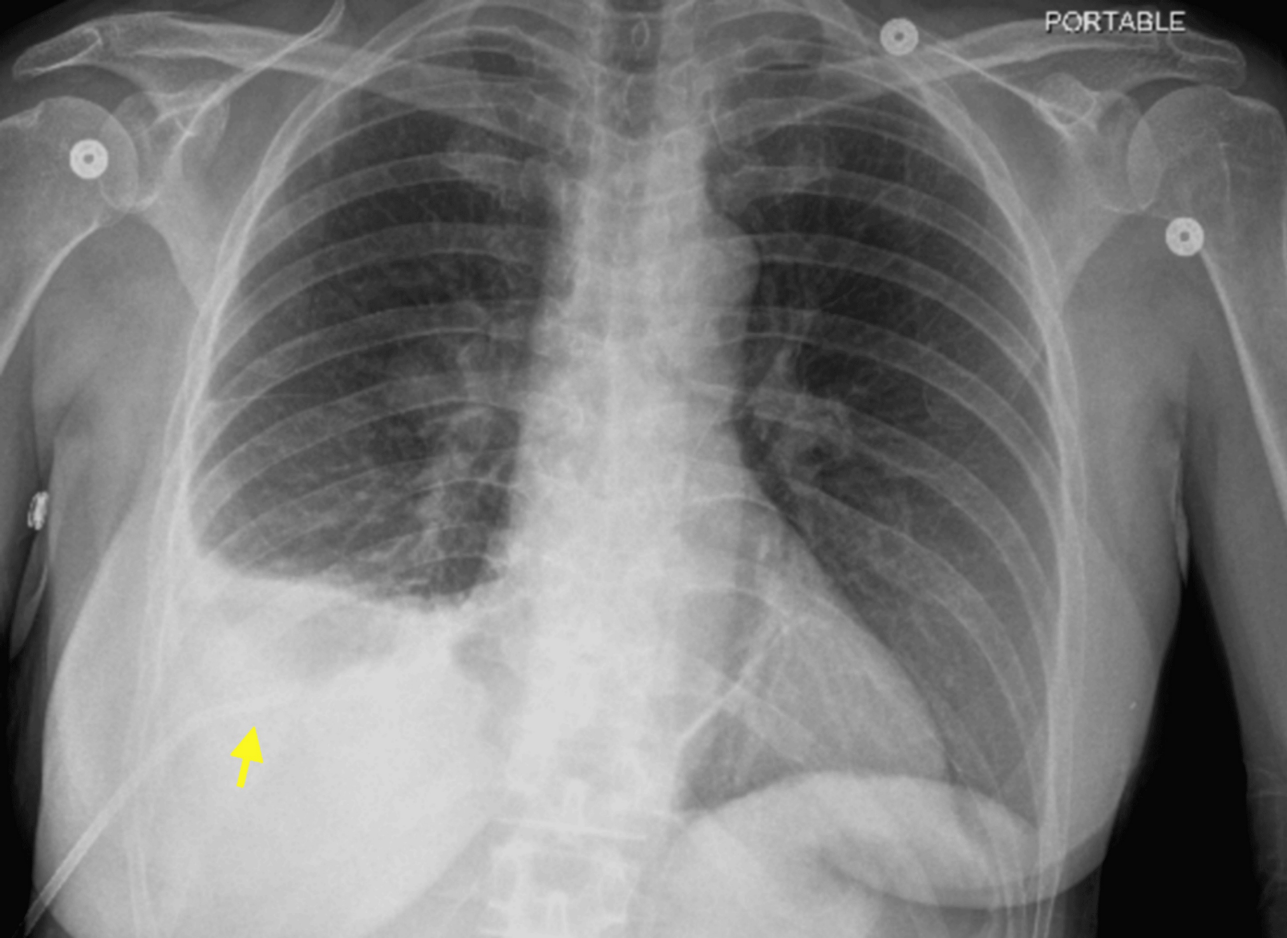
Persistent multiloculated right pleural effusion following chest tube placement and three days of intrapleural fibrinolytic therapy, demonstrating incomplete drainage despite intervention

She was started on intravenous vancomycin and cefepime for presumed healthcare-associated pneumonia. Ultrasound-guided thoracentesis was performed, followed by chest tube placement and three days of intrapleural fibrinolytic therapy with minimal drainage. Pleural fluid analysis was exudative (lactate dehydrogenase (LDH) 979 U/L, protein 4.5 g/dL) with negative cultures and cytology demonstrating no malignant cells. A follow-up CXR showed persistent pleural effusion (Figure 1).

On hospital day 3, she underwent bronchoscopy with bronchoalveolar lavage, which was negative for malignancy. Given persistent multiloculated pleural effusion and limited response to fibrinolytics, she was transferred to a tertiary care center on hospital day 4. Due to clinical stability, definitive surgical intervention was delayed, and she subsequently underwent robotic-assisted video-assisted thoracoscopic surgery (VATS) with decortication on hospital day 3 following transfer. Intraoperatively, there was an extensive inflammatory response with a dense pleural rind encasing the lung. Decortication was performed along with wedge resection of a right upper lobe lesion.

**Figure 2:**
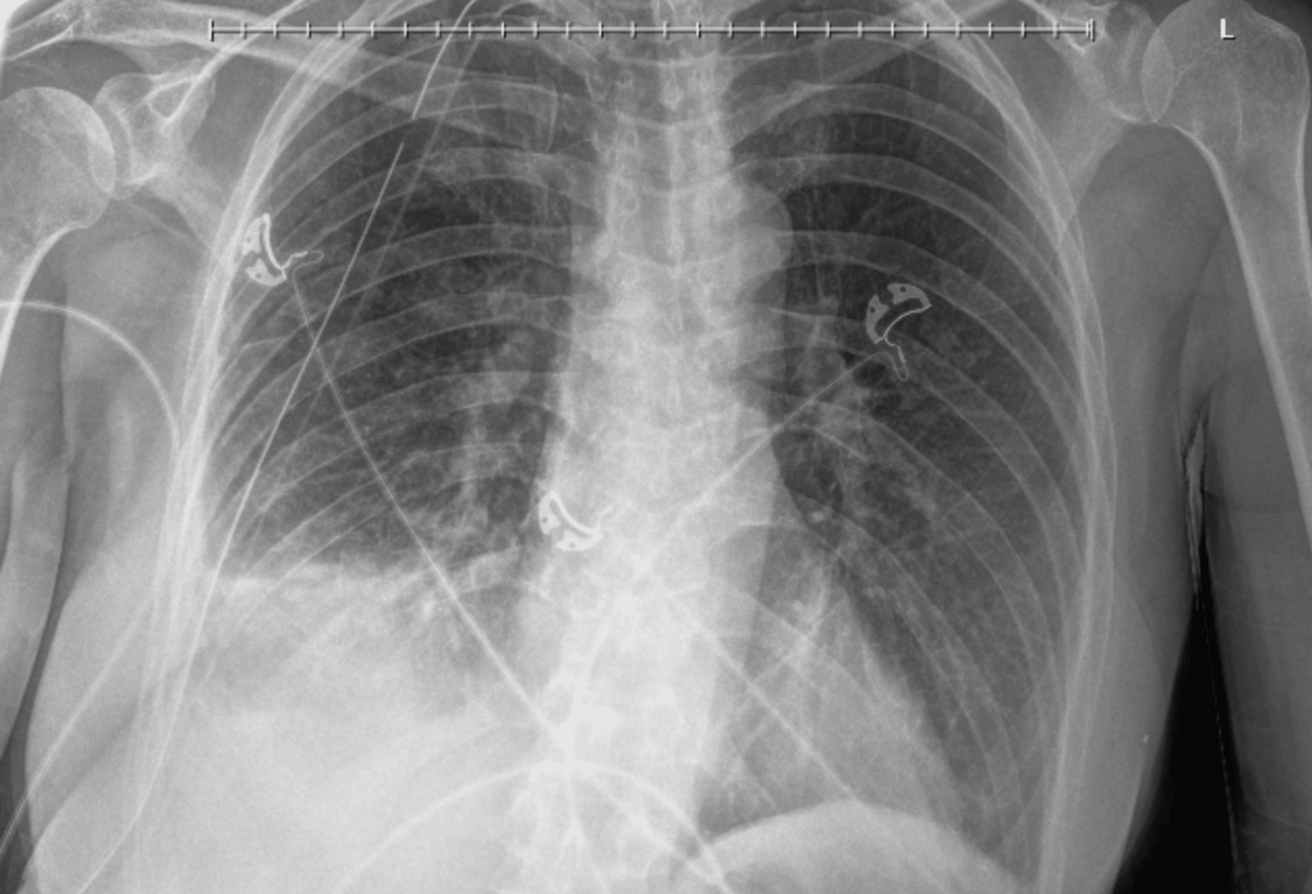
Near-complete resolution of the right pleural effusion following robotic-assisted VATS decortication with re-expansion of the right lung VATS: video-assisted thoracoscopic surgery

Pathologic evaluation of pleural rind, pleural mass, and lung tissue demonstrated fibrinopurulent inflammation and necrotic debris consistent with empyema. Immunohistochemical staining (pancytokeratin and CDX2) showed no evidence of metastatic appendiceal carcinoma. Pleural fluid and tissue cultures remained negative. Overall findings favored a reactive, nonmalignant empyema rather than metastatic disease. Postoperative imaging demonstrated improvement (Figure 2), and the patient was discharged in stable condition with outpatient oncology follow-up.

Following multidisciplinary tumor board discussion, management deviated from the standard treatment sequence. Given the patient’s recent abdominal surgery complicated by multiloculated pleural effusion and ongoing recovery, the decision was made to initiate systemic chemotherapy with capecitabine-oxaliplatin (CAPEOX) prior to right hemicolectomy, with plans for interval surgical resection. Pretreatment evaluation was notable for mild neutropenia, for which granulocyte colony-stimulating factor support was provided.

Treatment was complicated by transient chest pain attributed to coronary vasospasm, which improved with amlodipine. The patient subsequently completed three months of CAPEOX and underwent right hemicolectomy with ileocolostomy. Final surgical pathology demonstrated no residual tumor and 0/18 lymph nodes positive for malignancy (pT3 N0). Hyperthermic intraperitoneal chemotherapy (HIPEC) was deferred given the absence of peritoneal disease.

After multidisciplinary discussion and review of emerging data suggesting low recurrence risk following complete resection of localized GCA, adjuvant chemotherapy was discontinued, and the patient was transitioned to surveillance [12]. A summary of key clinical events is provided in Table 1.

**Table 1:** Timeline of major events CAPE-OX: capecitabine-oxaliplatin; HIPEC: hyperthermic intraperitoneal chemotherapy

Timeline of major events	
Day 0	Initial presentation for acute appendicitis
Day 7	Emergency room visit for progressive shortness of breath, fatigue, and rib pain
Day 11	Transfer to higher level facility for robotic decortication of pleural effusion
1 month	Oncology follow-up, initiation of CAPE-OX
4 months	Completion of 3 months of CAPE-OX. Surgical right hemicolectomy with peritoneal exploration, and deferral of HIPEC. Transition to surveillance management

## Discussion

This case of appendiceal GCA highlights the development of a multiloculated pleural effusion requiring surgical decortication, representing an atypical extraperitoneal manifestation. Large case series and systematic reviews describe metastatic spread of GCA primarily within the peritoneal cavity, including the ovaries, liver, and small bowel, with thoracic involvement rarely reported and typically occurring in the setting of advanced metastatic disease [6,7]. In contrast, this case demonstrates a multiloculated pleural process without evidence of thoracic metastasis.

Several etiologies were considered for the pleural effusion, including malignant, infectious, reactive inflammatory, and paraneoplastic processes. A malignant etiology was considered given the recent diagnosis of appendiceal GCA. However, pleural fluid cytology demonstrated no malignant cells, and pleural biopsy with immunohistochemical staining (pancytokeratin and CDX2) showed no evidence of metastatic appendiceal carcinoma. While pleural fluid cytology has known limitations in sensitivity, the absence of malignancy on both cytologic and tissue evaluation significantly lowers the likelihood of metastatic involvement.

An infectious etiology was also considered, particularly in the setting of recent hospitalization and imaging findings. However, pleural fluid cultures, including bacterial, mycobacterial, and fungal studies, were negative. Despite sterile cultures, the presence of fibrinopurulent material on pleural biopsy and intraoperative findings of a dense pleural rind are consistent with an empyema, which may reflect a sterile or partially treated infectious process.

A reactive inflammatory process was therefore favored as the most likely etiology. The pleural fluid was exudative by Light’s Criteria (LDH: 979 U/L; protein: 4.5 g/dL), and the patient had a recent history of appendiceal perforation with intra-abdominal inflammation. Nonmalignant pleural effusions have been described in association with intra-abdominal inflammatory processes due to diaphragmatic lymphatic drainage and increased capillary permeability, allowing translocation of inflammatory mediators across the pleuroperitoneal interface. In this context, the patient’s pleural effusion likely represents a reactive process that progressed to a multiloculated empyema requiring surgical intervention.

The standard management of GCA typically involves right hemicolectomy as first-line therapy, with adjuvant chemotherapy considered in patients with advanced-stage or recurrent disease [1,8]. In this case, surgical management was initially deferred due to the patient’s recent abdominal surgery, subsequent development of a multiloculated pleural effusion, and overall clinical condition. Additionally, early positron emission tomography/computed tomography (PET-CT) imaging was expected to have limited specificity in the immediate postoperative period due to increased fluorodeoxyglucose uptake in inflammatory and infectious processes, potentially confounding assessment of disease extent [13].

Given these factors, a multidisciplinary decision was made to initiate systemic chemotherapy with capecitabine-oxaliplatin (CAPEOX) prior to hemicolectomy. This approach allowed time for clinical recovery and more accurate staging prior to definitive surgical management. Ultimately, the patient underwent right hemicolectomy with no evidence of residual tumor or nodal involvement, allowing for avoidance of more aggressive interventions such as hyperthermic intraperitoneal chemotherapy (HIPEC).

Although the management strategy in this case deviated from conventional sequencing, it reflects the need for individualized treatment approaches in complex clinical scenarios. This case further highlights a rare extraperitoneal manifestation of GCA and underscores the importance of considering reactive pleural processes in the differential diagnosis of pleural effusions in patients with recent intra-abdominal pathology. Further study is needed to better characterize atypical presentations and management considerations in GCA.

## Conclusions

This case highlights an atypical presentation of appendiceal GCA complicated by a multiloculated pleural effusion requiring surgical intervention, as well as a deviation from the standard treatment sequence. While the pleural process was most consistent with a reactive inflammatory etiology in the setting of recent intra-abdominal pathology, a direct causal relationship with GCA cannot be definitively established. This case underscores the importance of maintaining a broad differential for pleural effusions in patients with recent intra-abdominal disease and highlights the role of multidisciplinary decision-making in guiding management in more complex clinical scenarios. This case may offer insight into similar situations where standard treatment pathways are not feasible, although conclusions are limited given this is a single case. Further investigation is warranted to better characterize potential thoracic complications associated with appendiceal tumors and to define optimal management strategies in these less common presentations.
